# The effect of milling frequency on a mechanochemical organic reaction monitored by in situ Raman spectroscopy

**DOI:** 10.3762/bjoc.13.216

**Published:** 2017-10-18

**Authors:** Patrick A Julien, Ivani Malvestiti, Tomislav Friščić

**Affiliations:** 1 Department of Chemistry, McGill University, Montreal, QC, Canada; 2 Departamento de Química Fundamental, Universidade Federal de Pernambuco, PE, Brazil

**Keywords:** green chemistry, mechanism, mechanochemistry, milling, monitoring, Raman spectroscopy

## Abstract

We provide the first in situ and real-time study of the effect of milling frequency on the course of a mechanochemical organic reaction conducted using a vibratory shaker (mixer) ball mill. The use of in situ Raman spectroscopy for real-time monitoring of the mechanochemical synthesis of a 2,3-diphenylquinoxaline derivative revealed a pronounced dependence of chemical reactivity on small variations in milling frequency. In particular, in situ measurements revealed the establishment of two different regimes of reaction kinetics at different frequencies, providing tentative insight into processes of mechanical activation in organic mechanochemical synthesis.

## Introduction

Over the past decade, mechanochemical reactions [1–4], i.e., chemical transformations induced or sustained through the application of mechanical force in the form of grinding, milling and shearing, have emerged as a highly versatile and general route to conduct chemical reactions in the absence of bulk solvents [2]. Indeed, the demonstrated versatility in organic [5–8], organometallic [9–10], pharmaceutical [11–12], supramolecular [13], metal-organic [14–15], and materials synthesis [16] has rendered mechanochemical reactions by ball milling or grinding as viable, highly environmentally-friendly alternatives to solution-based chemistry. Importantly, mechanochemistry provides not only a means to conduct chemical transformations of poorly soluble reagents [17], but also enables access to reactions that are difficult or even impossible to achieve in solution [18–20], and allows the synthesis of molecular targets that have so far been considered impossible to synthesize [21] or isolate [22].

However, in contrast to rapid expansion of applications of mechanochemistry, the mechanistic understanding of the underlying physicochemical process remains poor. It was only recently that significant effort was invested in understanding how fundamental environmental parameters, such as temperature, milling frequency, or sample-to-volume ratio [23–26] affect the course of organic mechanochemical reactions. A significant recent advance in mechanistic studies of mechanochemical reaction mechanisms was the introduction of techniques for in situ, real-time monitoring of ball milling processes [27], first through synchrotron X-ray powder diffraction (XRPD) [28–29], and later by Raman spectroscopy [30] or by a tandem technique combining these two techniques [31]. Whereas valuable mechanistic information on the course of a milling reaction can be obtained through stepwise, ex situ monitoring [32] based on periodically interrupting the milling process followed by sample extraction and analysis [33–34] such techniques can also lead to misleading results due to the sample either relaxing rapidly after milling [35] or reacting with surrounding atmosphere during preparation for analysis [36]. Such problems are additionally exacerbated in mechanochemistry of organic or metal-organic materials, readily activated through milling into transient, reactive amorphous phases. In contrast, real-time monitoring provides the opportunity to investigate the reaction course with time resolution in seconds, and without disrupting the milling process [31]. So far, the majority of real-time monitoring studies have focused on reactions of inorganic substances converting into metal-organic frameworks (MOFs) [17,28,37] or supramolecular reactions of cocrystallisation [38]. Real-time monitoring of an organic mechanochemical reaction was only recently reported by Tireli and co-workers, who utilized Raman spectroscopy to investigate how the choice of base influences the course of a base-catalysed nucleophilic substitution reaction [39].

**Scheme 1:**
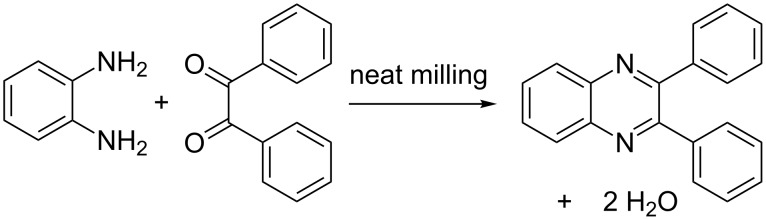
Milling synthesis of 2,3-diphenylquinoxaline from benzil and *ortho*-phenylenediamine [40].

Raman spectroscopy is particularly well-suited for monitoring and tracking organic reactions. It is a generally accessible and inexpensive, with an output based on changes to molecular structure rather than its crystallinity, offering a powerful tool for in situ studies of mechanochemical organic reactions that often proceed through amorphous or eutectic intermediates. We now report a Raman spectroscopy study of the effect of ball milling frequency on the course of a model organic transformation, the previously reported mechanochemical condensation of a diketone and a diamine to form an *N*-heteroacene [40]. We have utilized an in-house built setup for real-time Raman spectroscopy monitoring of the synthesis of 2,3-diphenylquinoxaline from benzil and *o*-phenylenediamine (Scheme 1). As the Raman signals of both reactants and the quinoxaline product can readily be distinguished, and the product can be obtained in high yield and purity by brief milling (less than an hour), we found this model system to be particularly appealing for mechanistic studies.

**Scheme 2:**
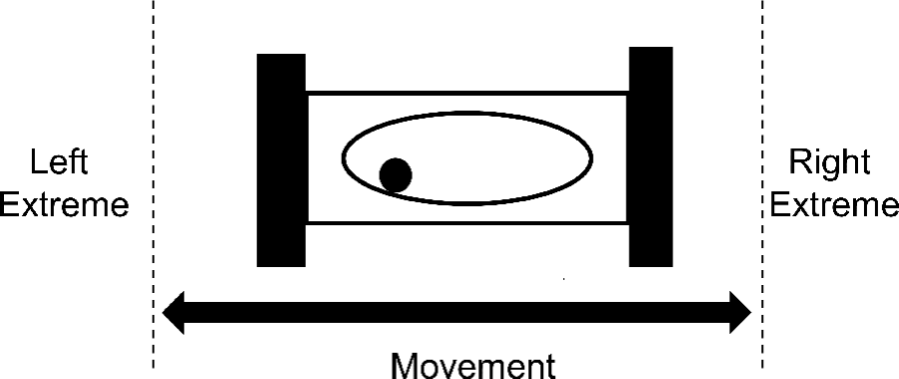
Movement of the milling jar and sample holder under milling conditions.

The milling frequency is one of the fundamental parameters of mechanochemical reactions conducted by ball milling, and for a vibratory shaker (mixer) ball mill it represents the number of full oscillations of the milling vessel (milling jar) per unit time along a curved path (Scheme 2). It is often used as a simple, primary assessment of the intensity of the milling process, and it affects the overall impact force, number and rate of impacts of milling media, as well as associated frictional heating.

Raman spectroscopy was recently utilized for a stepwise, ex situ assessment of the effect of milling frequency on the mechanochemical synthesis of a MOF from ZnO and imidazole in the presence of a small amount of *N*,*N*-dimethylformamide [41]. This study revealed reaction kinetics consistent with a 2nd order reaction rate law, rationalized through a “pseudo-fluid” reaction model in which the rate-determining factor is the frequency of reactive encounters between the particles. In contrast, ex situ gas chromatography studies of the Knoevenagel condensation between vanillin and barbituric acid in a planetary mill revealed a sigmoidal dependence of reaction yield with time [22]. Similarly, sigmoidal dynamics were detected by in situ XRPD monitoring of the formation of glycinium oxalate salts from γ-glycine and oxalic acid dihydrate [42]. Other examples of explorations of the effect of milling frequency on mechanochemical reactivity include aromatic substitution reactions [43] and the synthesis of nitrogen-doped titania [44], which have all revealed a non-linear relationship between milling frequency and reaction conversion.

## Results and Discussion

### In situ monitoring of the model condensation reaction

**Figure 1:**
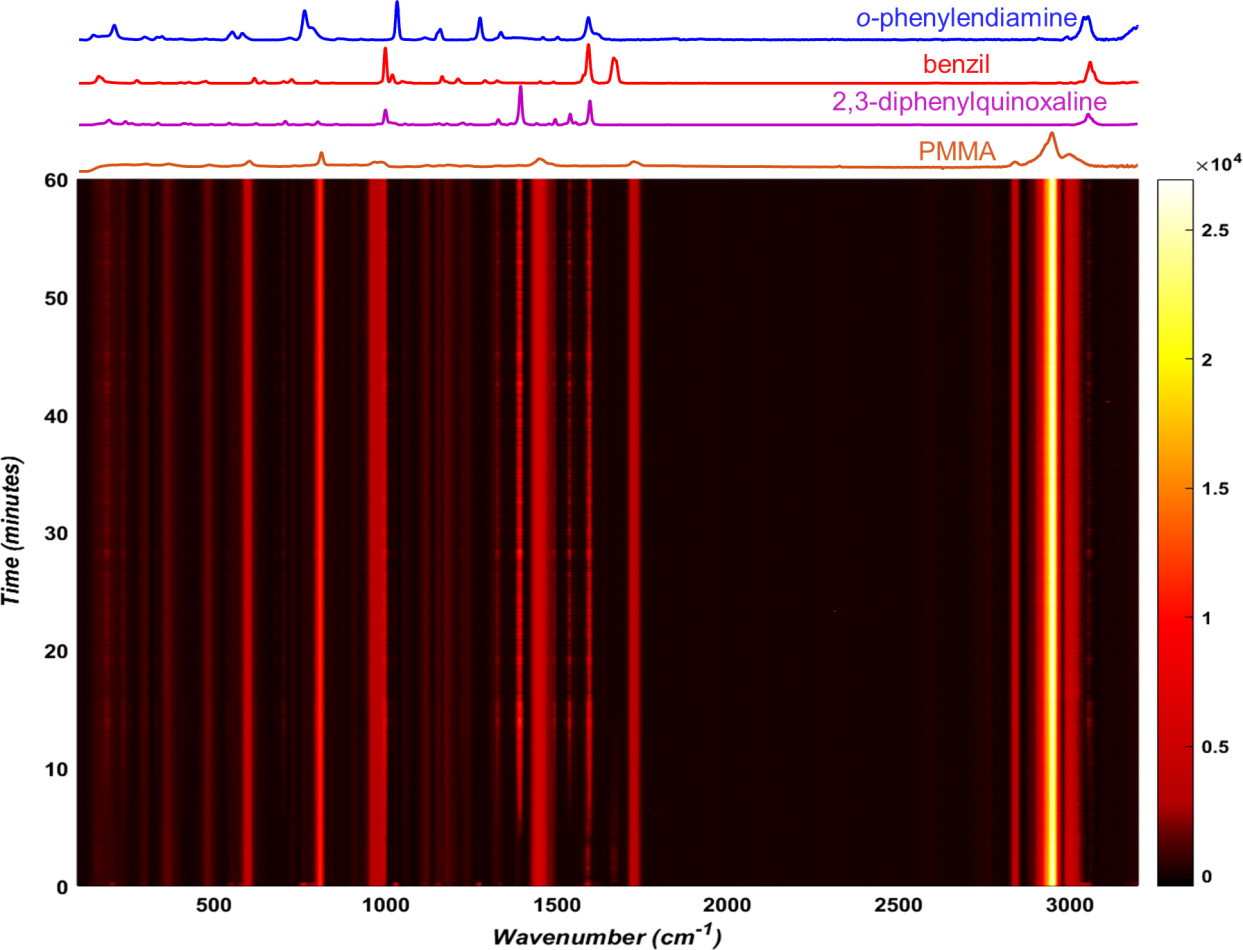
Time-resolved Raman spectrum for the double condensation of *o-*phenylenediamine and benzil to form 2,3-diphenylquinoxaline by milling at 30 Hz, with relevant spectra of reaction components and the PMMA milling jar shown above.

A preliminary investigation of the model condensation reaction was conducted by milling of *o*-phenylenediamine (108 mg, 1.0 mmol) with benzil (210 mg, 1.0 mmol) using a Retsch MM400 mixer mill operating at 30 Hz. The reaction mixture was placed in a 15 mL volume optically transparent poly(methyl metacrylate) (PMMA) jar, along with one zirconia ball of 10 mm diameter (ca. 3 grams weight). After 30 minutes milling, the analysis of the crude reaction product by ^1^H nuclear magnetic resonance (NMR) spectroscopy (see Supporting Information File 1) suggested quantitative conversion, with the presence of only trace impurities. Importantly, as the melting points of the starting materials and the product are considerably above room temperature (benzil: 94–96 °C; *o*-phenylenediamine: 100–102 °C; 2,3-diphenylquinoxaline: 125–127 °C) and no melting was observed upon grinding together of the two reactants, the formation of 2,3-diphenylquinoxaline is a good example of a solid-state reaction. Moreover, XRPD analysis of the crude reaction mixture after milling indicated that the product was crystalline (see Supporting Information File 1). Monitoring of the reaction in situ by Raman spectroscopy revealed the clear disappearance of reactant signals, as well as the emergence of strong signals of the product (Figure 1). Complete disappearance of reactant signals was observed in situ after ≈20 minutes milling, a timescale that is well suited for our study. Due to the significant scattering associated with collecting data through the 3 mm thick PMMA jar wall, all data were baseline corrected as described in the experimental section.

### Circumventing PMMA interference

**Figure 2:**
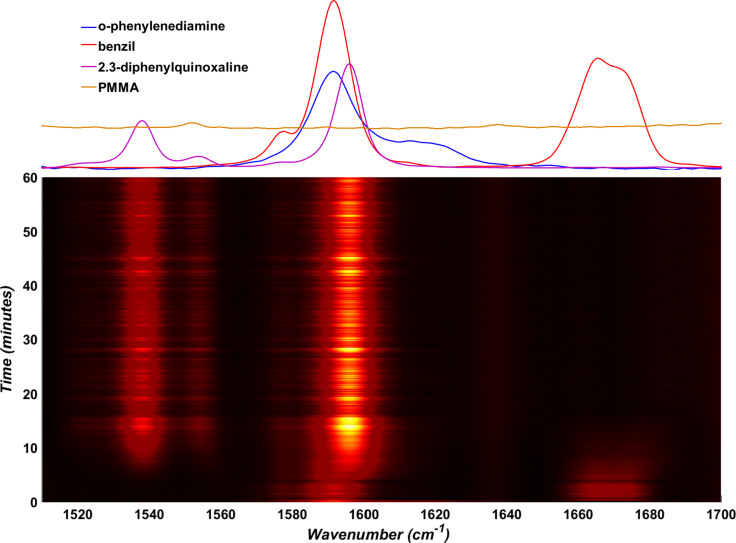
Section of the time-resolved Raman spectrum for the model mechanochemical reaction conducted at 30 Hz, that was selected for least-squares fitting. Normalized and baseline-corrected spectra for pure *o-*phenylenediamine (blue), benzil (red), 2,3-diphenylquinoxaline (purple) and an empty PMMA milling jar (yellow, offset for clarity) are shown above the time-resolved plot.

The milling jar wall produces a strong PMMA Raman signal which creates a strong background and interferes with in situ measurements of our reaction components. To minimize this effect, we focused our study on the spectral region between 1510 cm^−1^ and 1710 cm^−1^, where both starting materials and the product exhibit characteristic signals, and the PMMA spectrum is featureless (Figure 2).

### Fitting the dataset

A principal challenge associated with in situ monitoring of a milling reaction is the variation of the amount of sample in the beam due to the motion of the milling assembly. The resulting variations in the Raman signals of the sample and the scattering background affect the ability to monitor reaction progress, leading us to estimate the ratio of each component within the reaction mixture by a direct classical least-squares (CLS) approach based on experimentally obtained spectra of all scattering materials [45]. As the PMMA signal in the characteristic region between 1510–1700 cm^−1^ is sufficiently low to be neglected, this was limited to the spectra of the two starting materials, *o-*phenylenediamine and benzil, as well as the product 2,3-diphenylquinoxaline (Figure 2, top). The critical assumption in this approach is that all components are known and all spectral signals can be assigned to either the product or any of the reactants. Therefore, the calculated spectrum (C) can be described as a sum of pure component spectra *x_n_A_n_*, where *x_n_* is the contribution of each spectrum and *A_n_* is the spectrum of each pure component, with all components being known (Equation 1).

[1]
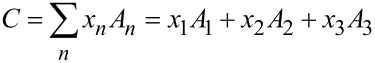


At the same time, the total sum of spectral contributions of all three reaction components must be equal to one, enabling the ratio of components to be calculated for each spectrum (Equation 2).

[2]
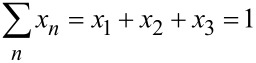


Variations in background scattering between all in situ collected spectra and the spectra of individual reaction components were accounted for by using the Sonneveld–Visser baseline correction algorithm [46]. In situ collected spectra were fitted as a sum of the normalized component spectra using a non-negative linear least squares algorithm (“lsqnonneg” in Matlab) which solves the fitting problem [47] of Equation 3:

[3]
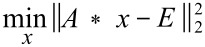


where *A* is a matrix containing the pure components spectra, *E* is the in situ obtained experimental spectrum, and *x* is a matrix of the mole fraction of each component, which satisfies *x* ≥ 0. Equation 3 provides the best values of *x* that minimize the difference between *A* * *x* and *E*.

**Figure 3:**
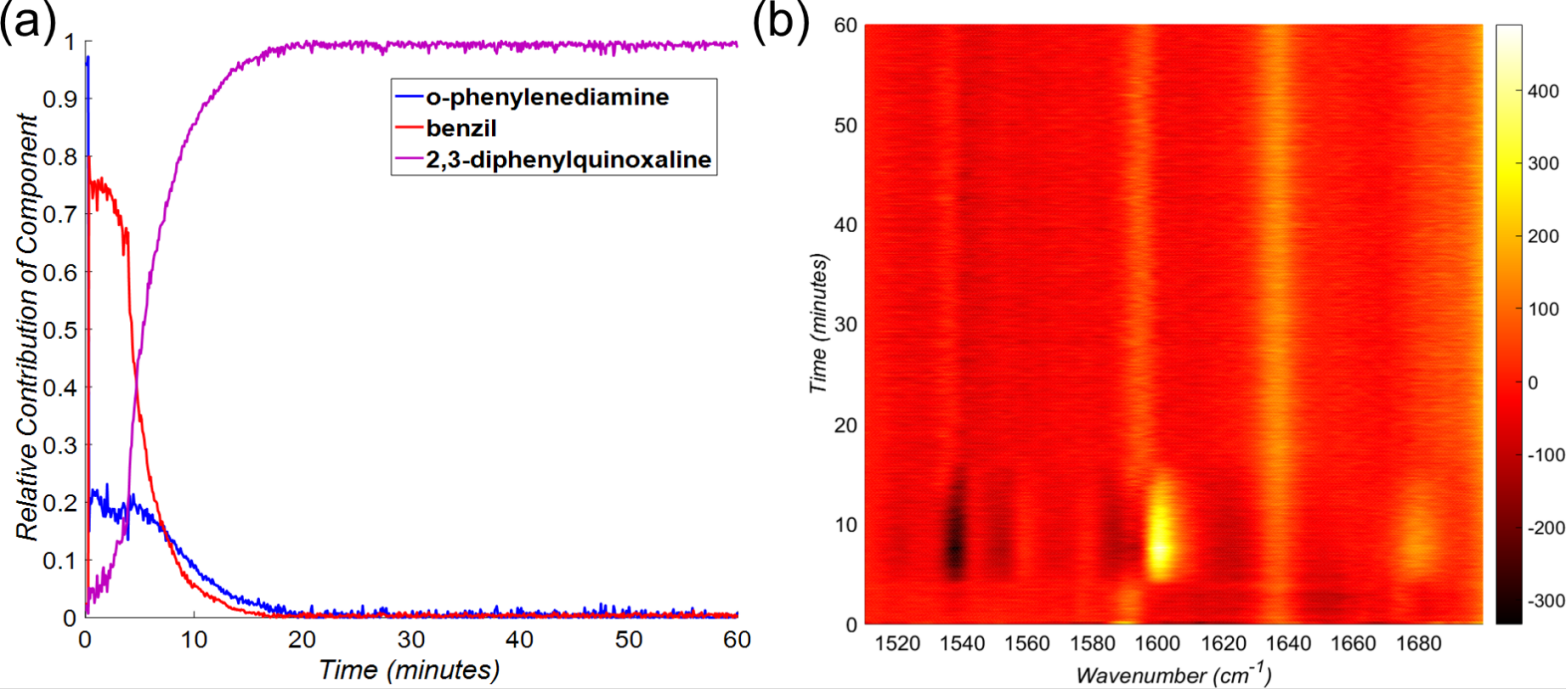
(Left) Estimated contribution of each component for each Raman spectrum over time of the synthesis of 2,3-diphenylquinoxaline at 30 Hz. (Right) Residual plot of the difference between experimental and estimated Raman spectra. In this case, this plot suggests an overestimation of 2,3-diphenylquinoxaline and an underestimation of *o*-phenylenediamine between ≈5 and ≈15 minutes. More information on the fitting can be found in Supporting Information File 1.

The described linear least-squares fitting procedure was applied to every spectrum in the in situ dataset and, following Equation 1 and Equation 2, enabled us to evaluate the relative spectral contribution of each reaction component *x_n_* (Figure 3).

It is important to note that the herein presented approach to data analysis assumes that the Raman spectra of individual reactants or products are not significantly affected by the degree of crystallinity or changes in the composition of the reaction mixture. While Raman scattering is expected to be directly proportional to the concentration of a particular molecular species [45], which suggests that the spectral contribution of a reaction component should also be directly proportional to its mole fraction, we have not yet calibrated this relationship. Accurate quantitative methods for analysing in situ Raman milling reactions are currently under development in our laboratory.

### The effect of milling frequency on the model reactions

**Figure 4:**
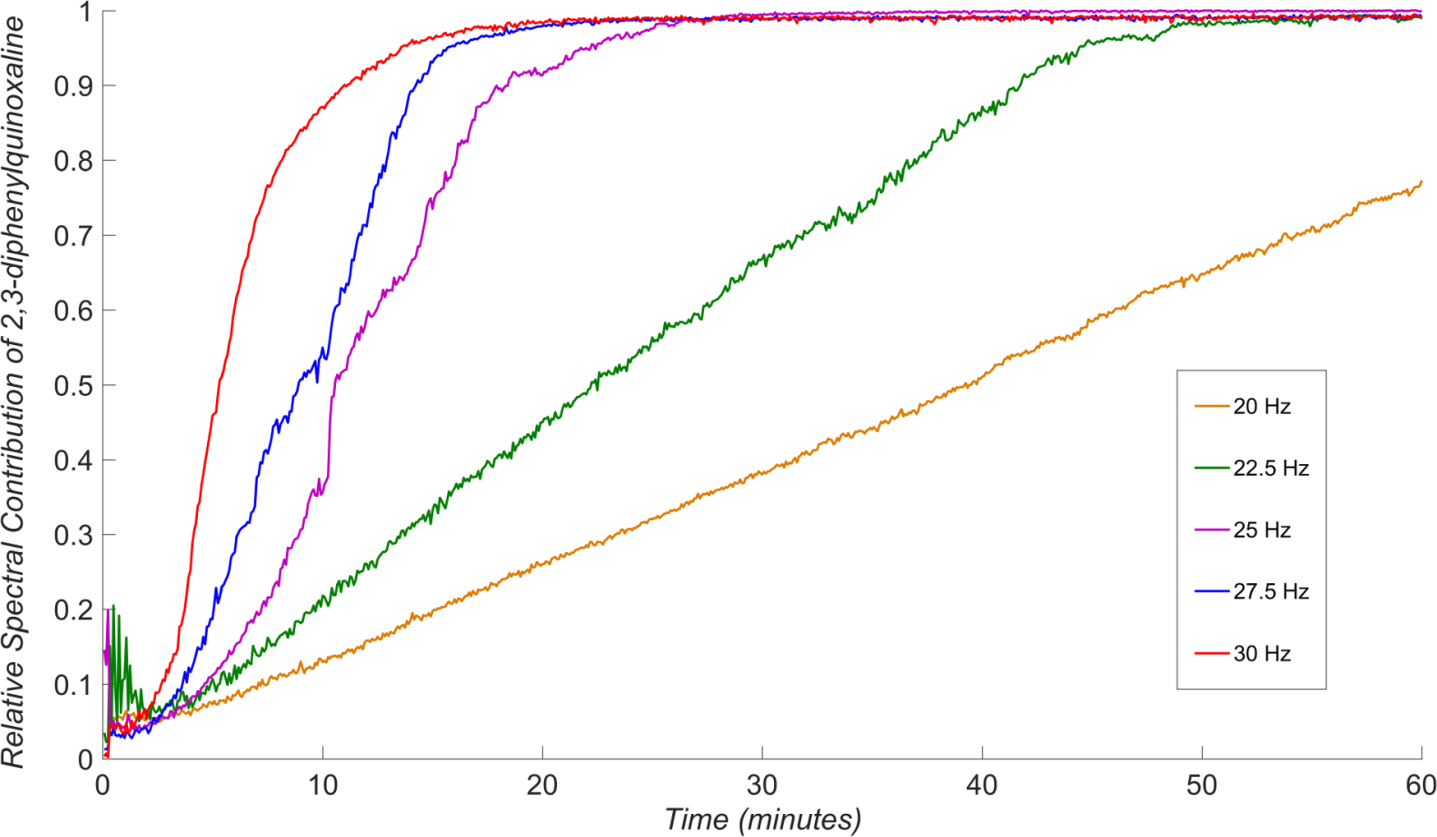
The effect of milling frequency on the milling condensation of benzil and *o-*phenylenediamine to form 2,3-diphenylquinoxaline, with data for each milling frequency averaged from a set of triplicate measurements. Variation close to the onset of milling may be due to poor initial homogeneity of the sample.

Having identified a suitable model reaction and an approach for the analysis of in situ reaction data, we were able to systematically explore the effect of milling frequency on the reaction rate. The systematic studies were conducted by measuring Raman spectra for chemical reactions that were, to the best of our ability, identical in all respects except the choice of milling frequency, i.e., the choice of milling media, the jar volume and material, the ball-to-sample weight ratio, and reactant batches were all kept constant. Specifically, we investigated the reaction behavior upon milling at 20 Hz, 22.5 Hz, 25 Hz, 27.5 Hz, and 30 Hz. For each of the frequencies, the measurements were performed in triplicate, and on the same day, in order to maximize reproducibility and minimize the variations in the reaction behaviour due to daily variation of ambient temperature or humidity. The final conversion for each experiment was verified by ^1^H NMR spectroscopy in solution (see Table S1 in Supporting Information File 1) and was found to be consistent with the in situ Raman spectroscopy data. Averaging the triplicate measurements of the time-dependent variation of product spectral contribution for each frequency (Figure 4) reveals remarkable sensitivity of the reaction rate on small changes in milling frequency.

**Figure 5:**
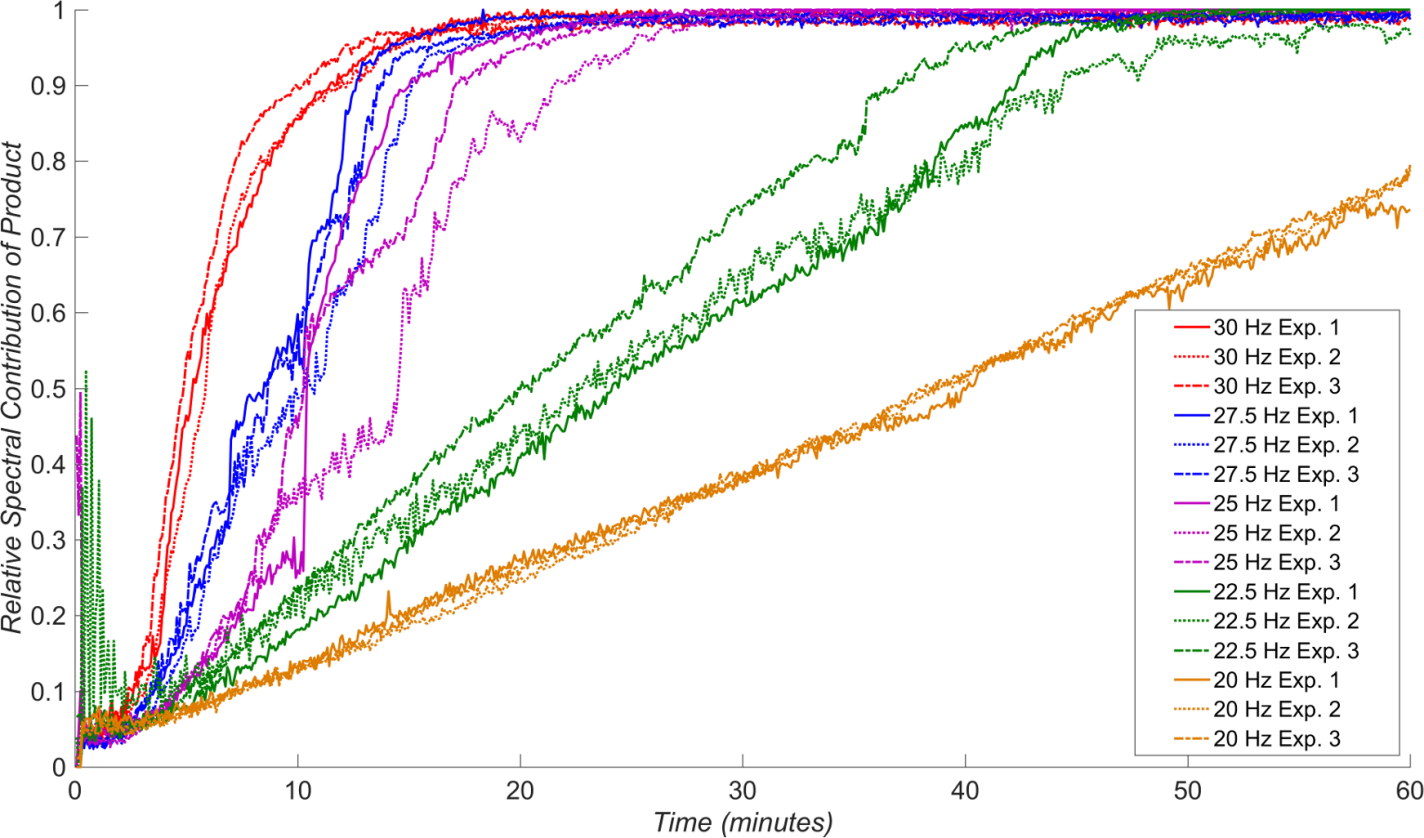
The reproducibility of varying milling frequency on the neat mechanochemical condensation of benzil and *o-*phenylenediamine, as measured by triplicate experiments.

The in situ monitoring data indicates that the reaction progress adopts a sigmoidal profile at milling frequencies higher than 25 Hz, which is consistent with the results of earlier ex situ studies of a Knoevenagel condensation reaction [22]. At milling frequencies below 25 Hz, however, the reaction appears to exhibits linear behavior. Further insight into the frequency-dependent behavior of our model reaction is obtained from the consistency of measurements within each set of triplicate in situ Raman scattering datasets for a given milling frequency (Figure 5). The individual datasets before averaging reveal that all measurements for a particular frequency are mutually consistent when milling at 30 Hz, 27.5 Hz, 22.5 Hz and 20 Hz.

At 25 Hz, however, the behavior of the reaction for each of the triplicate measurements was highly erratic and generally irreproducible. Overall, there is a clear difference in the kinetics of product formation when ball milling at 27.5 Hz and 30 Hz, compared to milling at frequencies of 20 Hz and 22.5 Hz, while milling at an intermediate frequency of 25 Hz led to irreproducible behavior. Tentatively, we interpret such switching between reactivity profiles by adopting the assumption that mechanochemical reactions proceed through the introduction of mechanically activated sites at which the reactions are facilitated, such as stacking faults and structural defects in general [48]. In such a scenario, different frequencies of milling are expected to lead to different levels of mechanical activation: at lower frequencies (i.e., 20 Hz or 22.5 Hz), the extent of mechanical activation is expected to be lower and product formation can progress at a similar rate to creation of novel activated sites. In contrast, at higher milling frequencies the rate of mechanical activation is much higher and product formation takes place in a highly activated environment, leading to a sigmoidal dependence of product formation with time. The above tentative explanation of our observations suggests that real-time Raman spectroscopy studies could offer an opportunity to directly probe the nature of mechanical activation underlying mechanochemical reactivity. Importantly, the proposed explanation is also consistent with different modes of ball motion during milling, as lower frequencies are known to favor rolling and shearing motion, whereas higher ones should lead to a greater number of more energetic mechanical impacts [25,49].

### Milling frequency vs temperature

**Figure 6:**
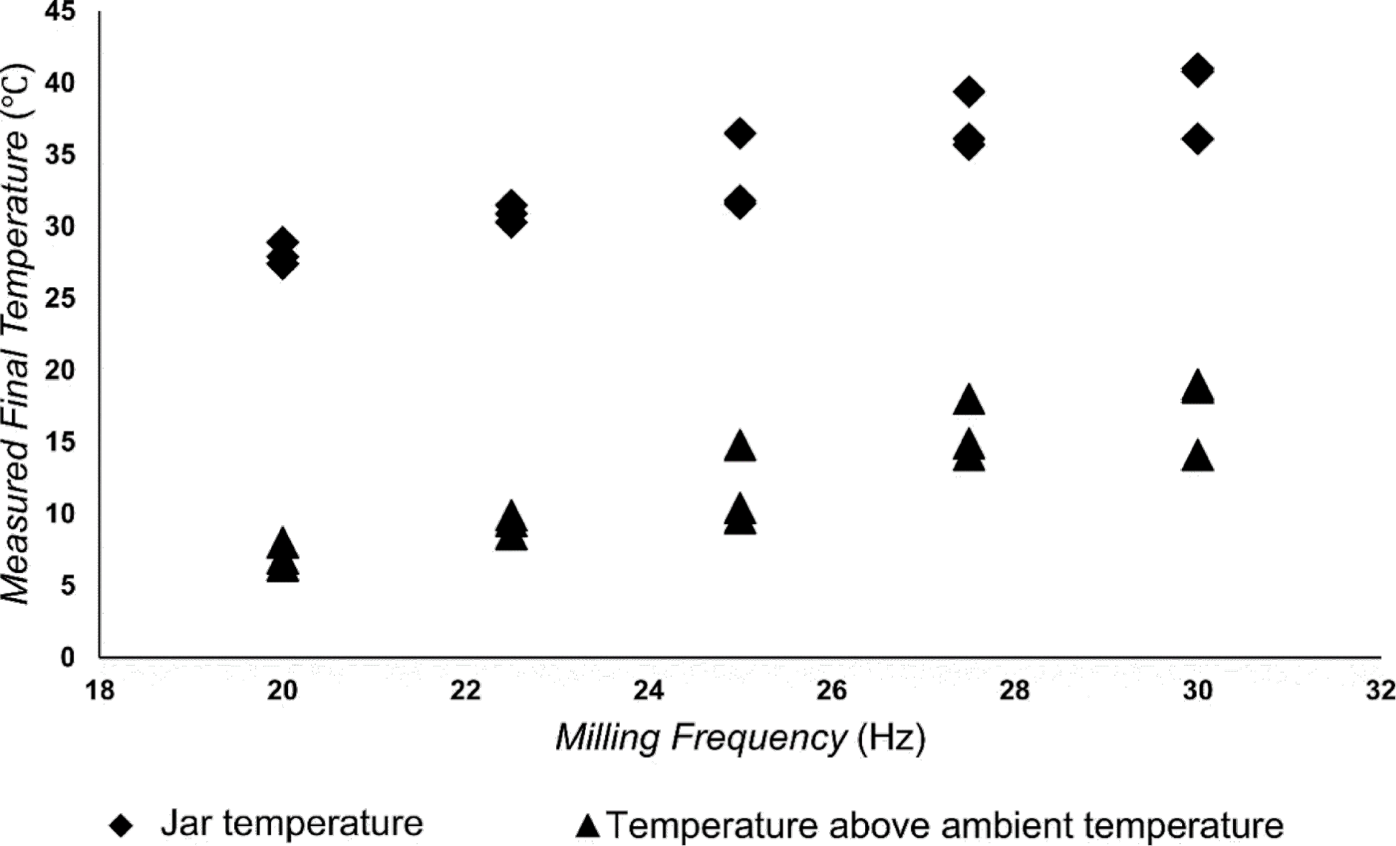
The effect of milling frequency on the internal jar temperature measured immediately after reaction completion.

One of the challenges in exploring the effects of milling frequency on mechanochemical reactivity is the increase in temperature of milling jars due to frictional heating [50–51]. Due to such heating effects, an increase in milling frequency should lead not only to greater mechanical activation, e.g., through impact and structure deformation, but also to an increase in reaction rate [52]. In order to evaluate the thermal effect associated with each of herein explored ball milling frequencies, we have also measured the temperature of the internal jar wall immediately after milling, revealing a potentially linear relationship between milling frequency and jar temperature (Figure 6). Importantly, the measured temperature never exceeded 45 °C, and was never higher than 19 °C above the ambient temperature. Although the observed temperature increases are generally not very large, they might be relevant for the observed variation of reaction kinetics with milling frequency, especially as a recent variable-temperature in situ PXRD study has demonstrated that mechanochemical reaction rates can be highly sensitive to temperature [52].

## Conclusion

In conclusion, we have utilized an in-house Raman spectroscopy setup to conduct real-time, in situ monitoring of the progress of a model mechanochemical organic reaction at different ball milling frequencies. The methodologies for real-time monitoring of mechanochemistry by ball milling have only recently been introduced and have so far been employed largely in studies of metal-organic or organic materials (e.g., model pharmaceutical cocrystals). The herein presented proof-of-principle study is the first to investigate in real time how the milling frequency, which is one of the fundamental parameters of mechanochemical reactivity, affects an organic transformation. Our results reveal high sensitivity of a carefully selected model mechanochemical reaction to the milling frequency, and establishment of clearly different regimes of reaction kinetics depending on the frequency. At lower frequencies, the model reaction exhibits a largely linear profile, resembling pseudo-zero order reaction kinetics, whereas increasing the frequency lead to a switch to apparently sigmoidal behavior. While these observations can tentatively be rationalized by different levels of mechanical activation of the reaction mixture at different frequencies, validating such an explanation requires further and quantitative studies. Nevertheless, we believe that the apparent ability of a mechanochemical reaction to switch between different regimes of chemical kinetics in response to minor changes in milling frequency is an important observation not only in the context of organic mechanochemistry, and may even be of importance in reconciling differences in recently reported in situ and ex situ studies of mechanochemical reactivity [22,41–42,53].

## Experimental

### Chemicals

Benzil (98%) was purchased from Aldrich Chemical. *o-*Phenylenediamine (98%) was purchased from Alfa Aesar. Both were used without further purification.

### Milling reactions and characterization

The double condensation was conducted by milling 210 mg of benzil (1.0 mmol) and 108 mg of *o-*phenylenediamine (1.0 mmol) with a single zirconia ball of 10 mm diameter (ca. 3 grams weight) in a 15 mL poly(methyl methacrylate) (PMMA) optically transparent milling jar, using a Retsch^®^ MM400 mixer mill. For all real-time reaction monitoring, reactions were monitored using a RamanRxn1™ analyzer by Kaiser Optical Systems Inc. every 5 seconds using a 785 nm laser. Spectra were dark and intensity corrected using the Holograms^®^ software package before being processed. The products of these reactions were analyzed without purification. The identity of the final product was confirmed through ^1^H and ^13^C NMR in CDCl_3_ using a 500 MHz AVIIIHD 500 Bruker spectrometer. Infrared spectra were collected on a Bruker Vertex 70 FT-IR Platinum ATR, while X-ray powder diffraction patterns were collected on a Proto Manufacturing AXRD Benchtop Powder Diffractometer using Ni-filtered Cu K_α_ radiation. The conversion for each solid-state reaction was evaluated after milling using ^1^H NMR spectroscopy conducted in CDCl_3_ on a 300 MHz Varian Mercury spectrometer. The ambient temperature was measured using a digital thermometer by VWR and the internal jar temperature was acquired immediately after milling finished using a Mastercraft Temperature Reader with Digital Display and Laser Pointer (accuracy ±2 °C).

## Supporting Information

File 1 Experimental part.
