# Supplementary material for: The effect of milling frequency on a mechanochemical organic reaction monitored by in situ Raman spectroscopy
Source: Beilstein J Org Chem. 2017 Oct 18;13:2160–8. doi: 10.3762/bjoc.13.216 (PMC5669241; doi:10.3762/bjoc.13.216)
Supplement: File 1 — Experimental part. [file Beilstein_J_Org_Chem-13-2160-s001.pdf]

Supporting Information

for

The effect of milling frequency on a mechanochemical organic reaction monitored by in situ Raman spectroscopy

Patrick A. Julien[1], Ivani Malvestiti[1,2] and Tomislav Friščić[1]*

Address: [1]Department of Chemistry, McGill University, Montreal, QC, Canada and [2]Departamento de Química Fundamental, Universidade Federal de Pernambuco, PE, Brazil

Email: Tomislav Friščić - tomislav.friscic@mcgill.ca

* Corresponding author

Experimental part

Table of Contents

1. Selected Raman spectra of pure components S2
2. Experimental setup S2
3. Product characterization S3
4. Spectroscopic data and fitting plots for all experiments S6

1. Selected Raman spectra of pure components

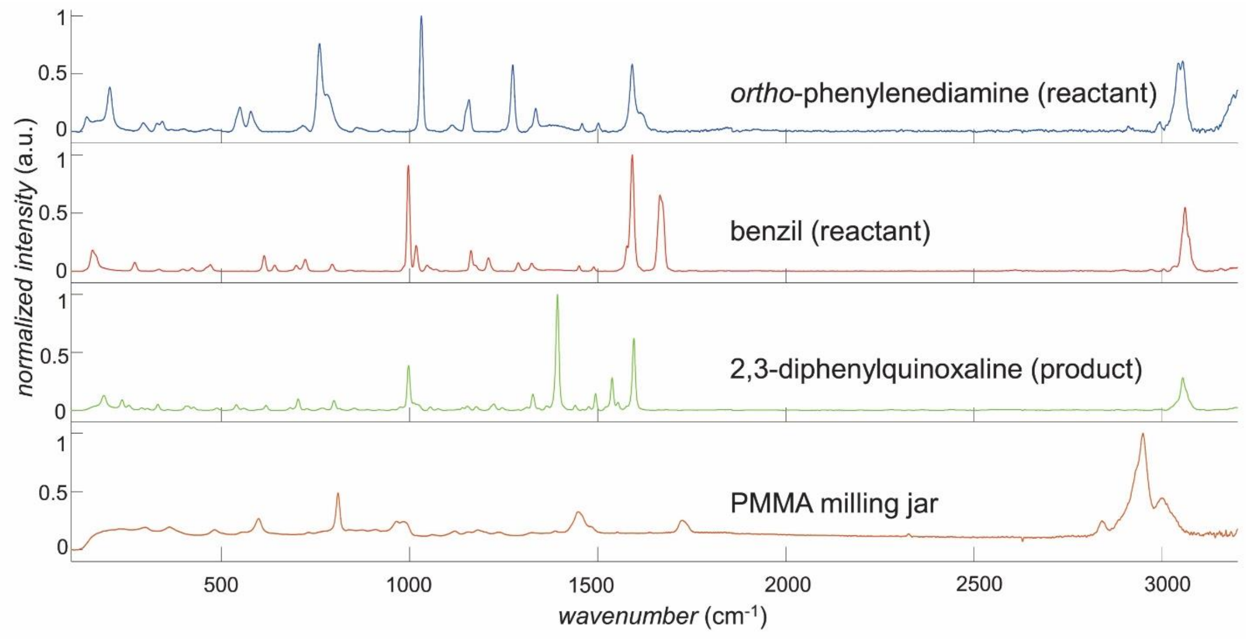

Figure S1: Relevant Raman spectra (top to bottom): reactant *ortho*-phenylenediamine; reactant benzil; product 2,3-diphenylquinoxaline and an empty PMMA milling jar.

2. Experimental setup

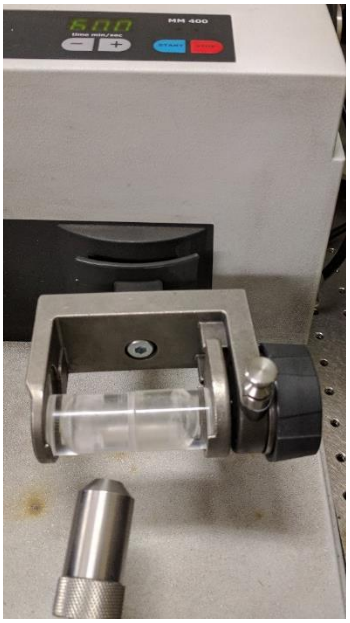

Figure S2: Picture of the experimental setup, showing a 15 mL volume PMMA jar mounted on a milling station of the Retsch MM400 mixer mill and the tip of the Raman spectroscopy probe.

3. Product characterization

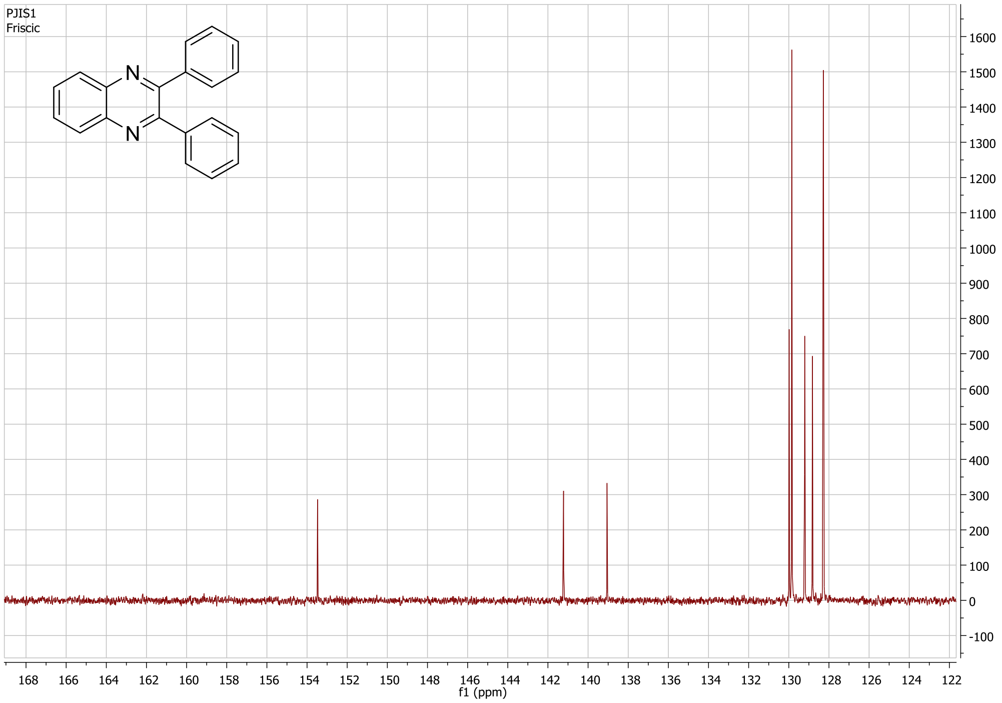

Figure S3: ^{1}H NMR spectrum of the crude 2,3-diphenylquinoxaline product obtained by mechanochemical reaction conducted at 30 Hz (30 Hz, Exp. 1), recorded in $CDCl_3$. The spectrum reveals only trace impurities and matches the spectrum previously reported in the *Spectral Database for Organic Compounds (SDBS)*, SDBS No. 32951HSP-48-683.

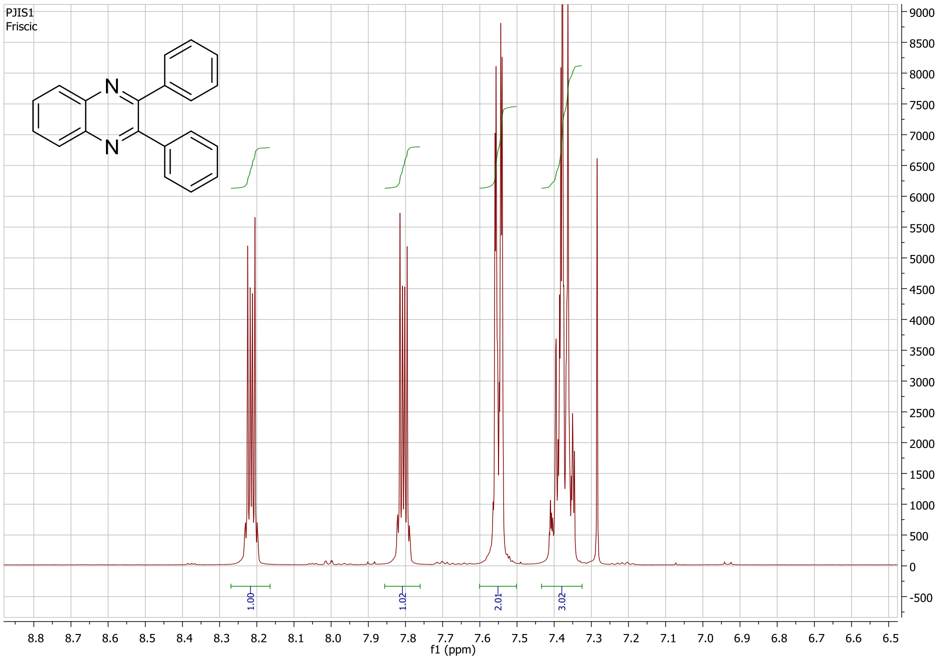

Figure S4: ^{13}C NMR spectrum of the crude 2,3-diphenylquinoxaline product obtained by mechanochemical reaction conducted at 30 Hz (30 Hz, Exp. 1), recorded in $CDCl_3$. The spectrum reveals only trace impurities and matches the spectrum previously reported in the *Spectral Database for Organic Compounds (SDBS)*, SDBS No. 32951CDS-10-500

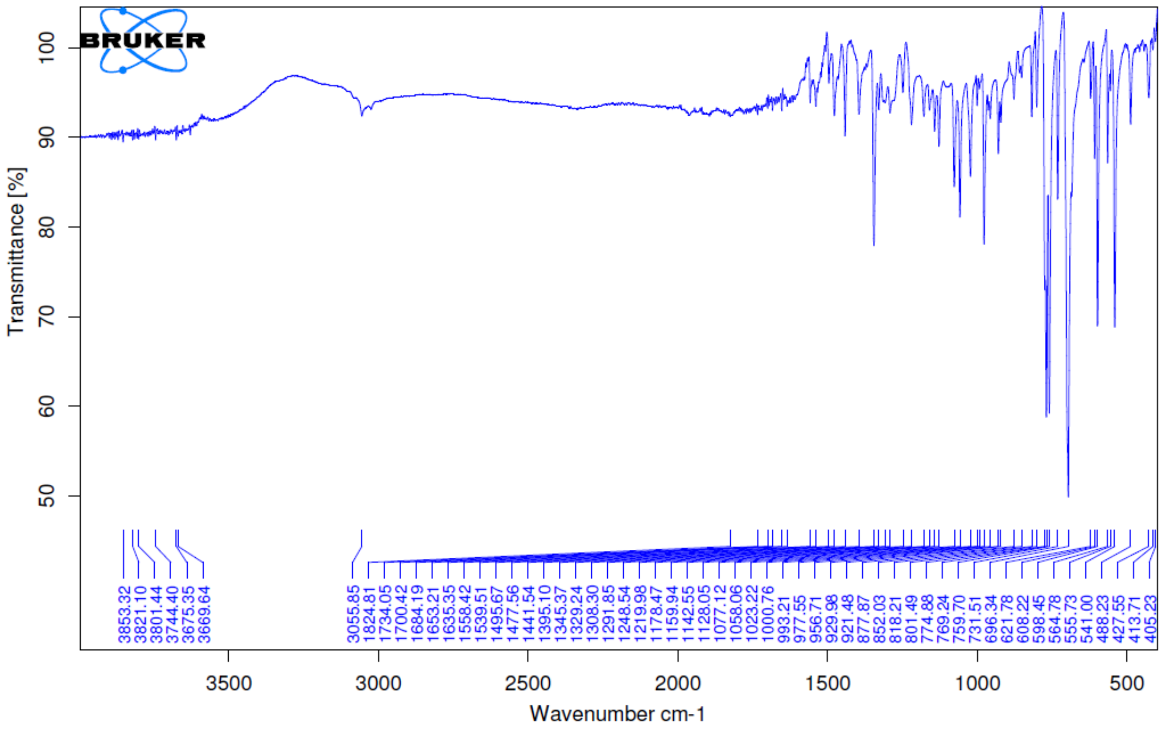

Figure S5: Fourier-transform infrared attenuated total reflectance (FTIR-ATR) spectrum of the crude 2,3-diphenylquinoxaline product obtained by mechanochemical reaction conducted at 30 Hz (30 Hz, Exp. 1). The spectrum matches the spectrum previously reported in the *Spectral Database for Organic Compounds (SDBS)*, SDBS No. 32951 IR-NIDA-74153.

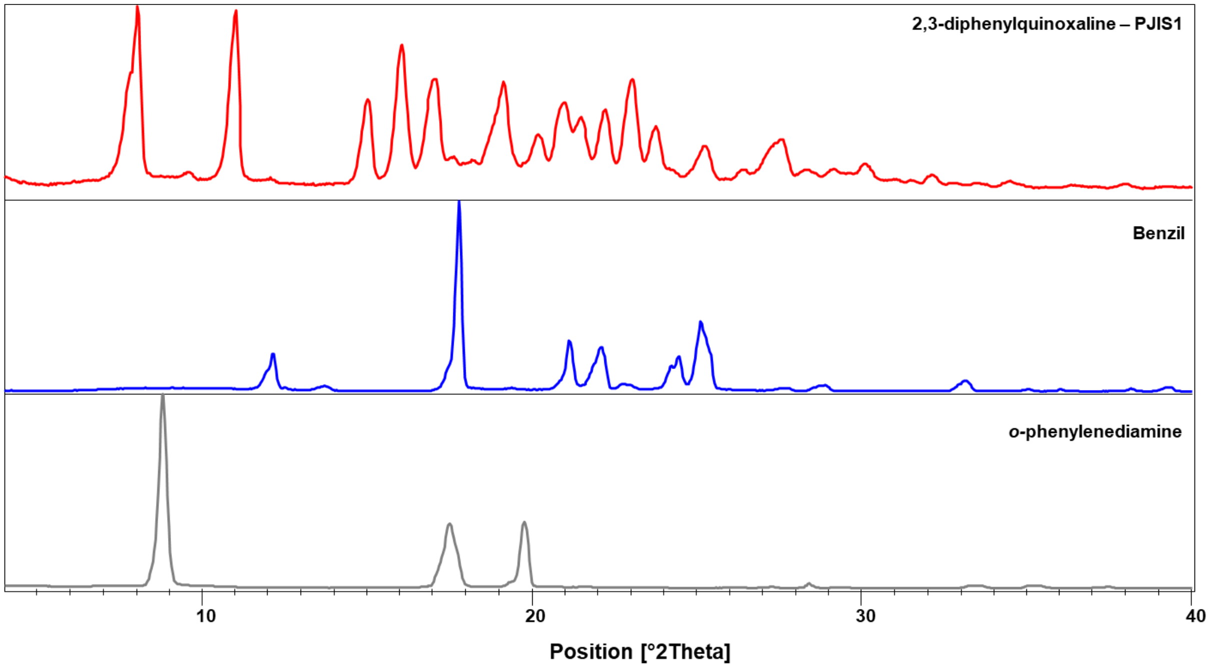

Figure S6: Example powder X-ray diffraction patterns for the mechanochemical synthesis of 2,3-diphenylquinoxaline (top to bottom): the crude product of the reaction conducted by ball milling at 30 Hz (30 Hz, Exp. 1); benzil reactant and reactant *ortho*-phenylenediamine.

Table S1: Reaction conversions, based on integration of ^{1}H NMR spectra of crude milling products. If starting material could not be detected, conversion is listed as 99+%

Reaction Number	Frequency (Hz) and Experiment #	Conversion (%)
1	**30, Exp. 1**	**99+**
2	**30, Exp. 2**	**99+**
3	**30, Exp. 3**	**99+**
4	**27.5, Exp. 1**	**99+**
5	**27.5, Exp. 2**	**99+**
6	**27.5, Exp. 3**	**97.0**
7	**25, Exp. 1**	**99+**
8	**25, Exp. 2**	**98.0**
9	**25, Exp. 3**	**99+**
10	**22.5, Exp. 1**	**99+**
11	**22.5, Exp. 2**	**99+**
12	**22.5, Exp. 3**	**99+**
13	**20, Exp. 1**	**79.5**
14	**20, Exp. 2**	**74.5**
15	**20, Exp. 3**	**83.0**

4) Spectroscopic data and fitting plots for all experiments

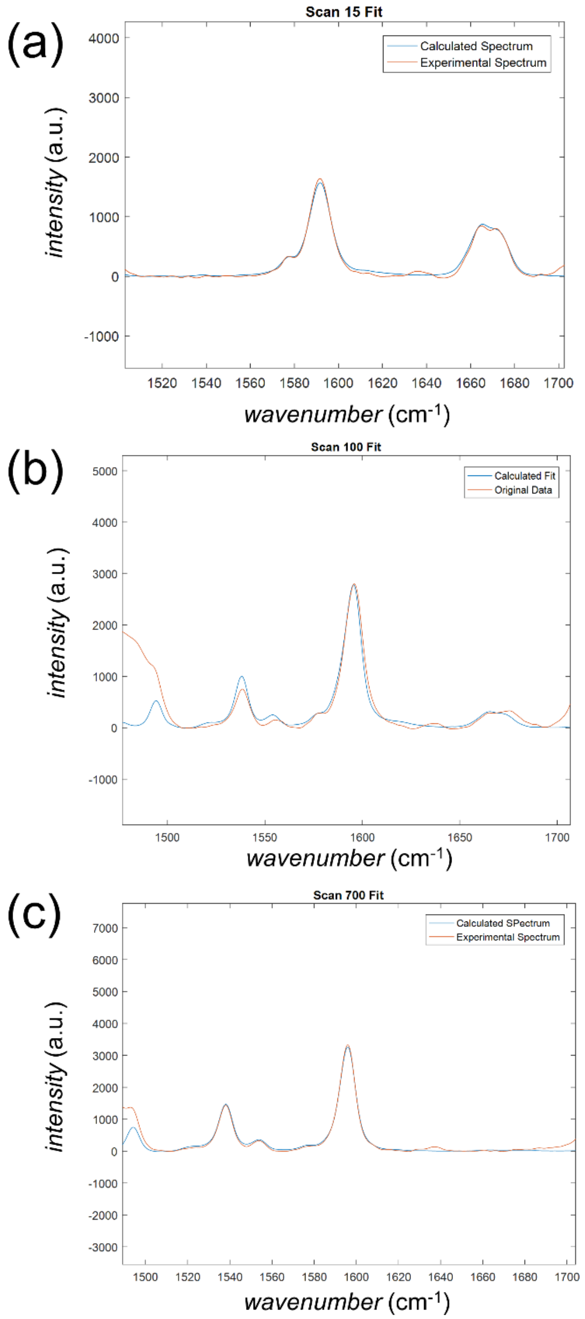

Figure S7: Comparison of experimental and calculated spectra for Experiment 1, 30 Hz (the complete dataset and residuals for this experiment are presented in the article, main paper): (a) scan #15 (after 75 seconds milling); (b) scan #100 (after 500 seconds milling) and (c) scan #700 (after 3500 seconds milling).

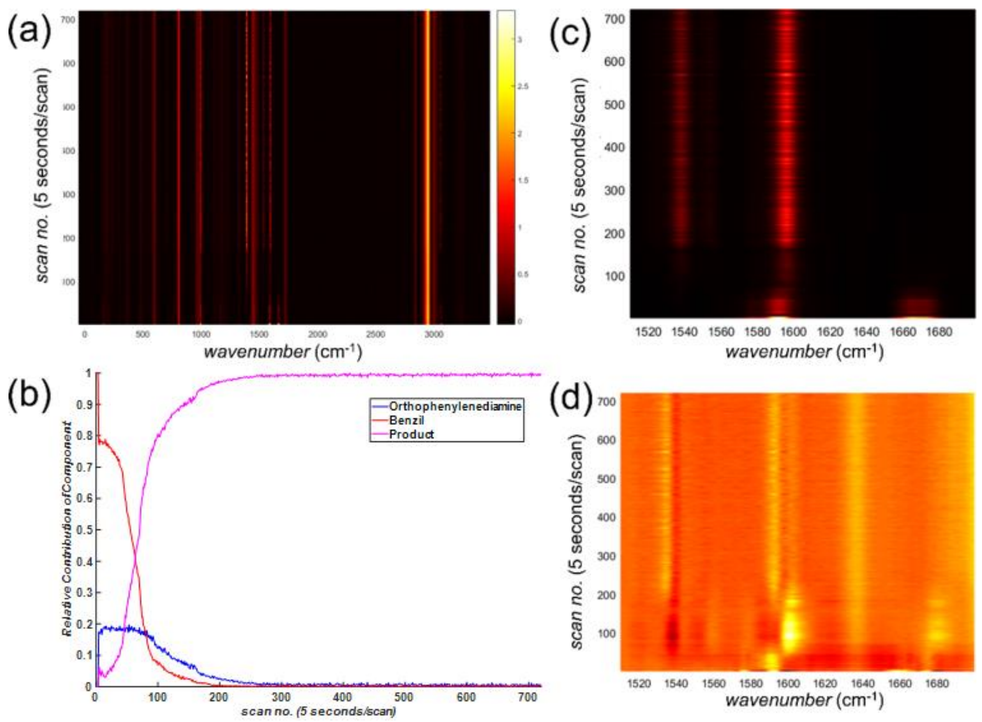

Figure S8: Data for Experiment 2, 30 Hz: (a) the entire time-resolved Raman spectrum for the second experiments conducted by milling at 30 Hz; (b) the associated time-dependent change in spectral contributions of the product (purple) and the two reactants (red and blue); (c) section of the spectrum selected for least-squares fitting and (d) the final residual spectrum.

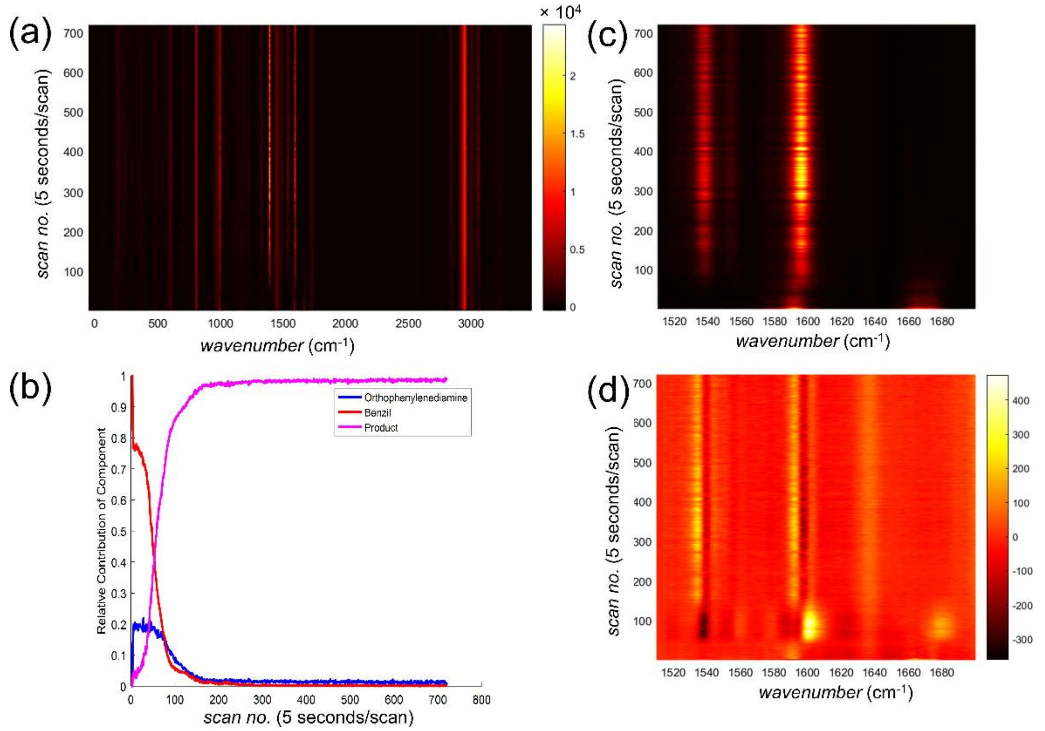

Figure S9: Data for Experiment 3, 30 Hz: (a) the entire time-resolved Raman spectrum for the third experiment conducted by milling at 30 Hz; (b) the associated time-dependent change in spectral contributions of the product (purple) and the two reactants (red and blue); (c) section of the spectrum selected for least-squares fitting and (d) the final residual spectrum.

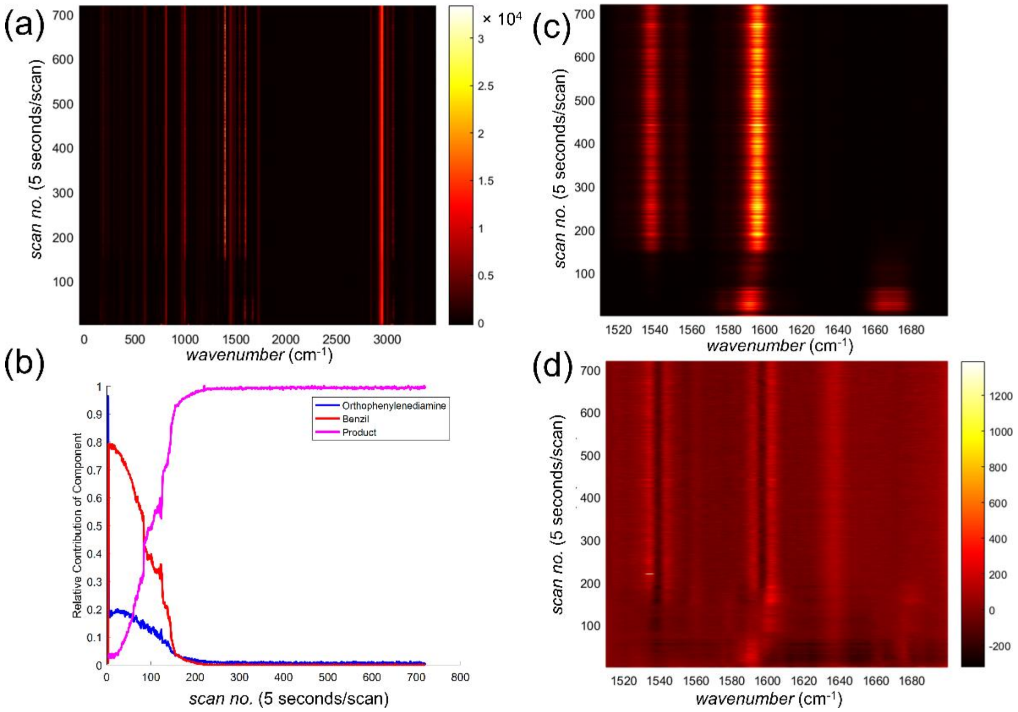

Figure S10: Data for Experiment 1, 27.5 Hz: (a) the entire time-resolved Raman spectrum for the first experiment conducted by milling at 27.5 Hz; (b) the associated time-dependent change in spectral contributions of the product (purple) and the two reactants (red and blue); (c) section of the spectrum selected for least-squares fitting and (d) the final residual spectrum.

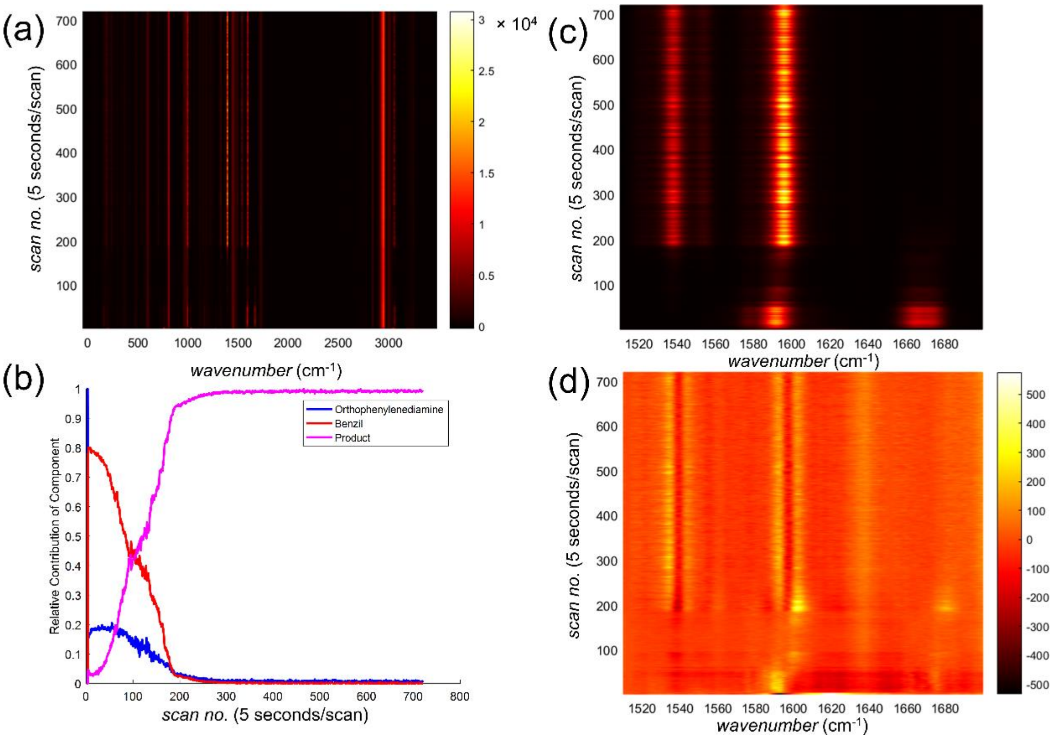

Figure S11: Data for Experiment 2, 27.5 Hz: (a) the entire time-resolved Raman spectrum for the second experiment conducted by milling at 27.5 Hz; (b) the associated time-dependent change in spectral contributions of the product (purple) and the two reactants (red and blue); (c) section of the spectrum selected for least-squares fitting and (d) the final residual spectrum.

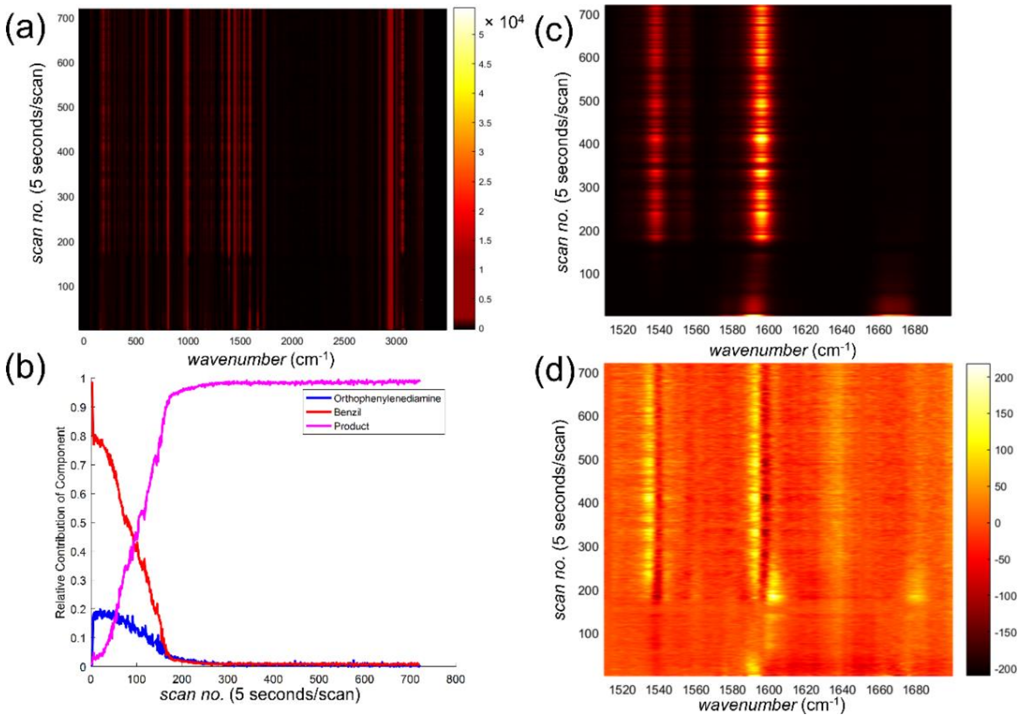

Figure S12: Data for Experiment 3, 27.5 Hz: (a) the entire time-resolved Raman spectrum for the third experiment conducted by milling at 27.5 Hz; (b) the associated time-dependent change in spectral contributions of the product (purple) and the two reactants (red and blue); (c) section of the spectrum selected for least-squares fitting and (d) the final residual spectrum.

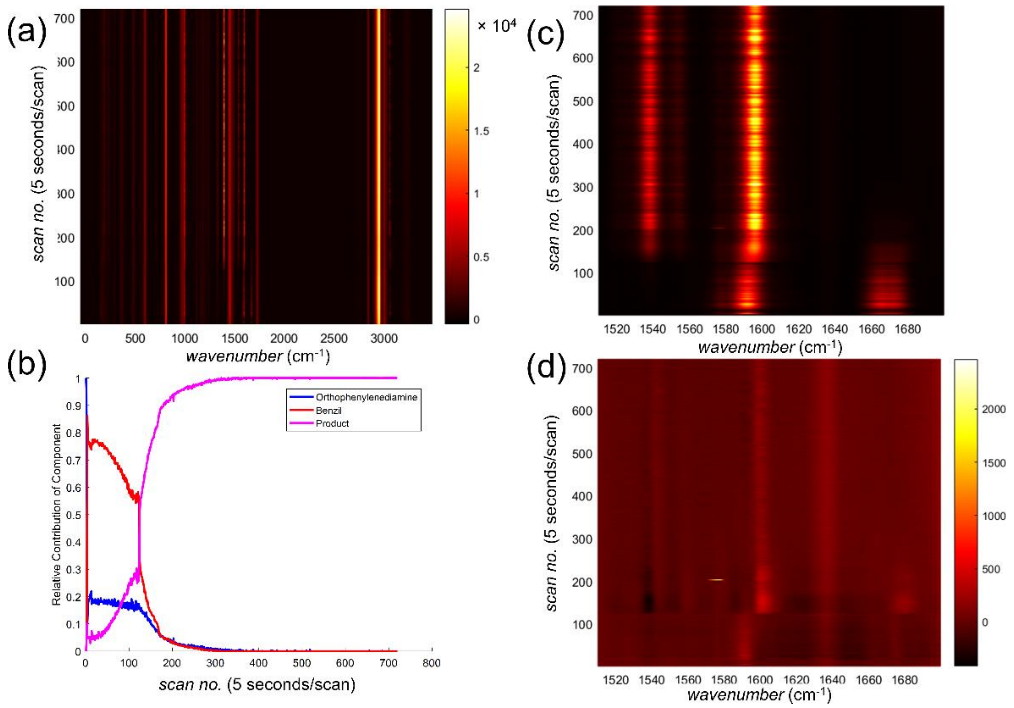

Figure S13: Data for Experiment 1, 25 Hz: (a) the entire time-resolved Raman spectrum for the first experiment conducted by milling at 25 Hz; (b) the associated time-dependent change in spectral contributions of the product (purple) and the two reactants (red and blue); (c) section of the spectrum selected for least-squares fitting and (d) the final residual spectrum.

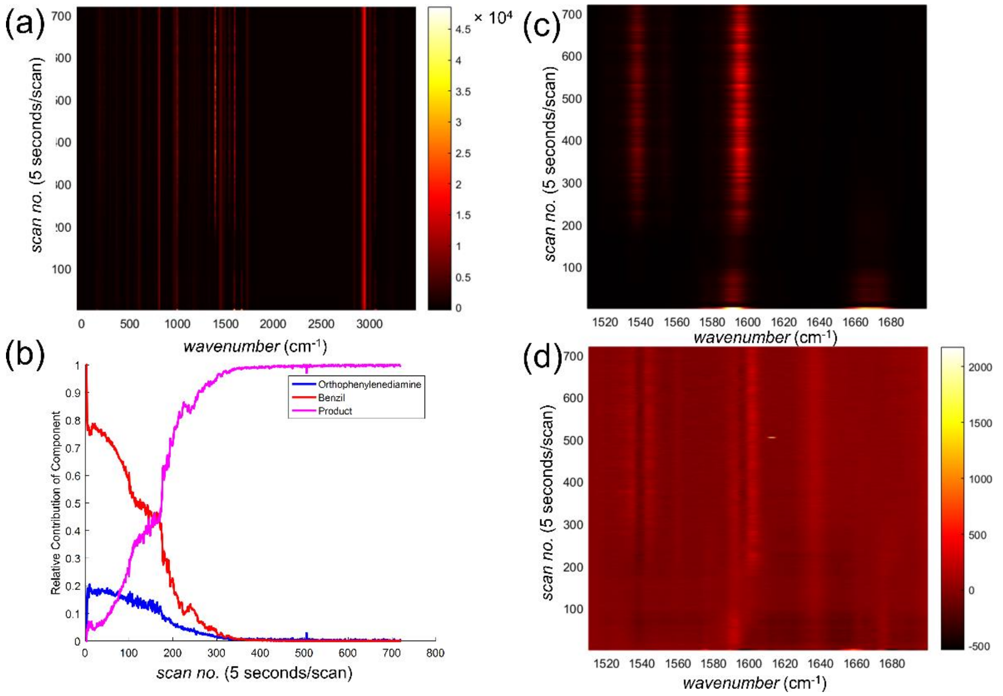

Figure S14: Data for Experiment 2, 25 Hz: (a) the entire time-resolved Raman spectrum for the second experiment conducted by milling at 25 Hz; (b) the associated time-dependent change in spectral contributions of the product (purple) and the two reactants (red and blue); (c) section of the spectrum selected for least-squares fitting and (d) the final residual spectrum.

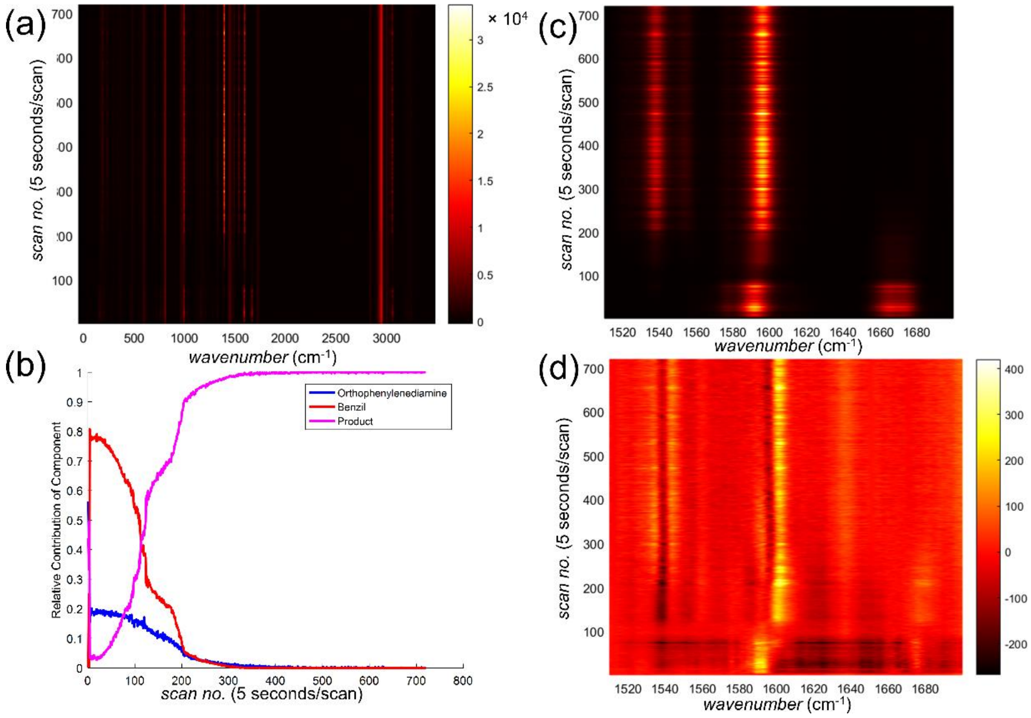

Figure S15: Data for Experiment 3, 25 Hz: (a) the entire time-resolved Raman spectrum for the third experiment conducted by milling at 25 Hz; (b) the associated time-dependent change in spectral contributions of the product (purple) and the two reactants (red and blue); (c) section of the spectrum selected for least-squares fitting and (d) the final residual spectrum.

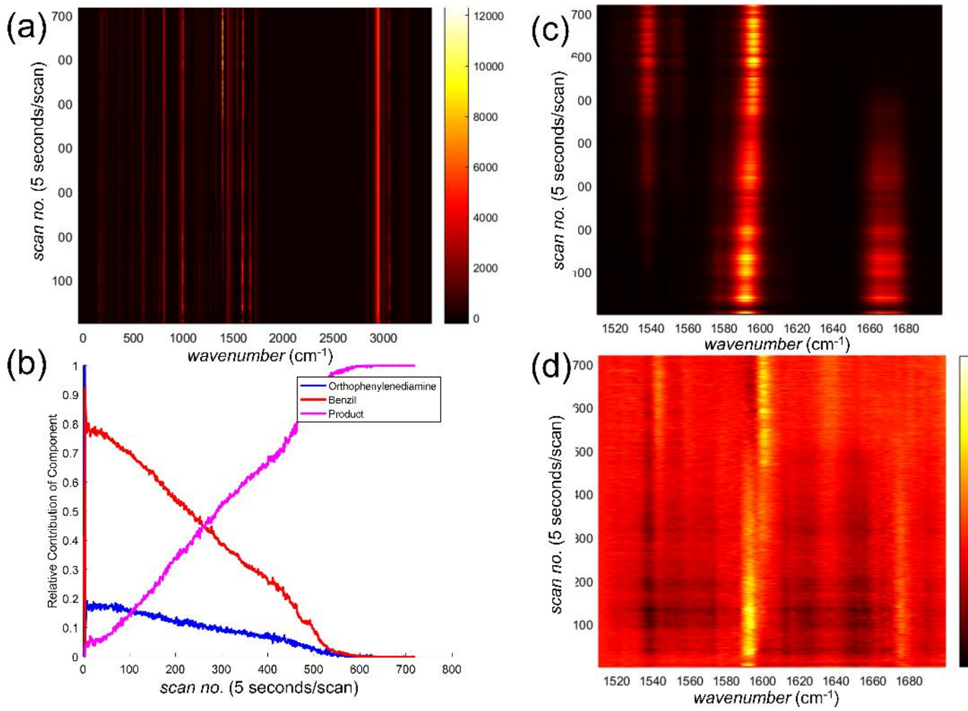

Figure S16: Data for Experiment 1, 22.5 Hz: (a) the entire time-resolved Raman spectrum for the first experiment conducted by milling at 22.5 Hz; (b) the associated time-dependent change in spectral contributions of the product (purple) and the two reactants (red and blue); (c) section of the spectrum selected for least-squares fitting and (d) the final residual spectrum.

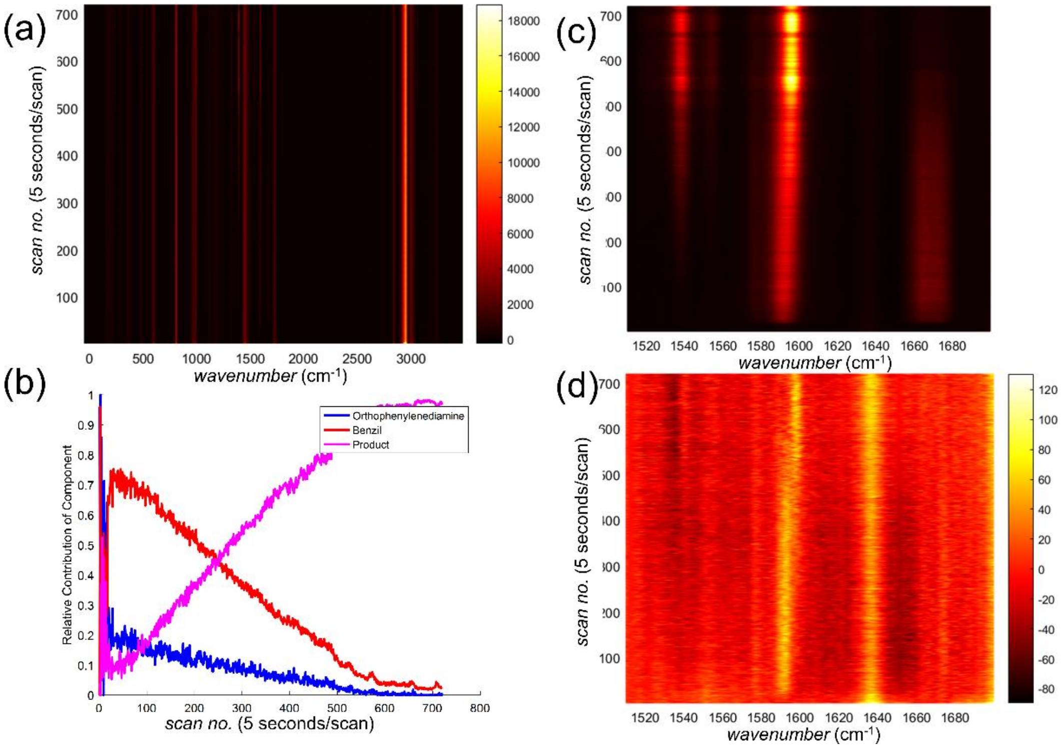

Figure S17: Data for Experiment 2, 22.5 Hz: (a) the entire time-resolved Raman spectrum for the second experiment conducted by milling at 22.5 Hz; (b) the associated time-dependent change in spectral contributions of the product (purple) and the two reactants (red and blue); (c) section of the spectrum selected for least-squares fitting and (d) the final residual spectrum.

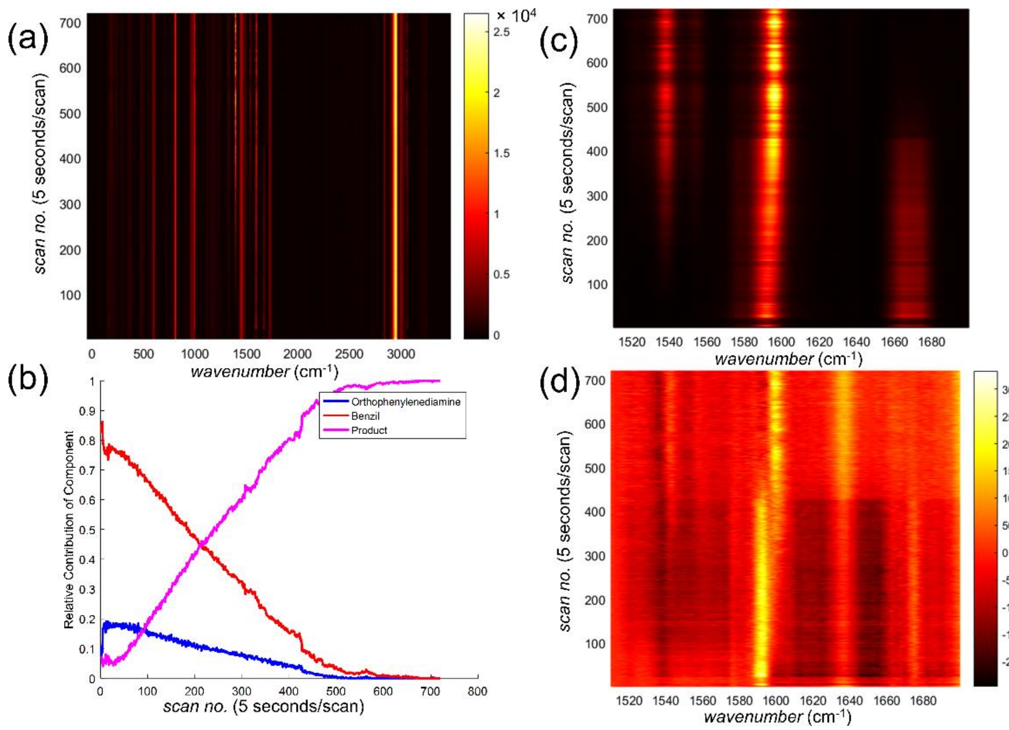

Figure S18: Data for Experiment 3, 22.5 Hz: (a) the entire time-resolved Raman spectrum for the third experiment conducted by milling at 22.5 Hz; (b) the associated time-dependent change in spectral contributions of the product (purple) and the two reactants (red and blue); (c) section of the spectrum selected for least-squares fitting and (d) the final residual spectrum.

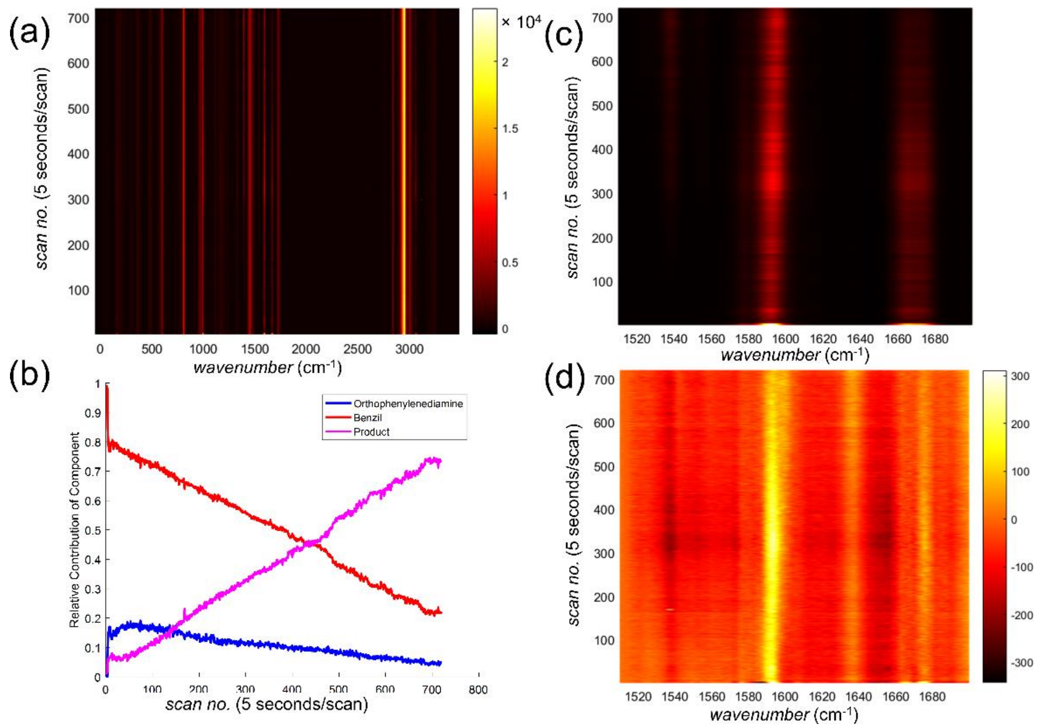

Figure S19: Data for Experiment 1, 20 Hz: (a) the entire time-resolved Raman spectrum for the first experiment conducted by milling at 20 Hz; (b) the associated time-dependent change in spectral contributions of the product (purple) and the two reactants (red and blue); (c) section of the spectrum selected for least-squares fitting and (d) the final residual spectrum.

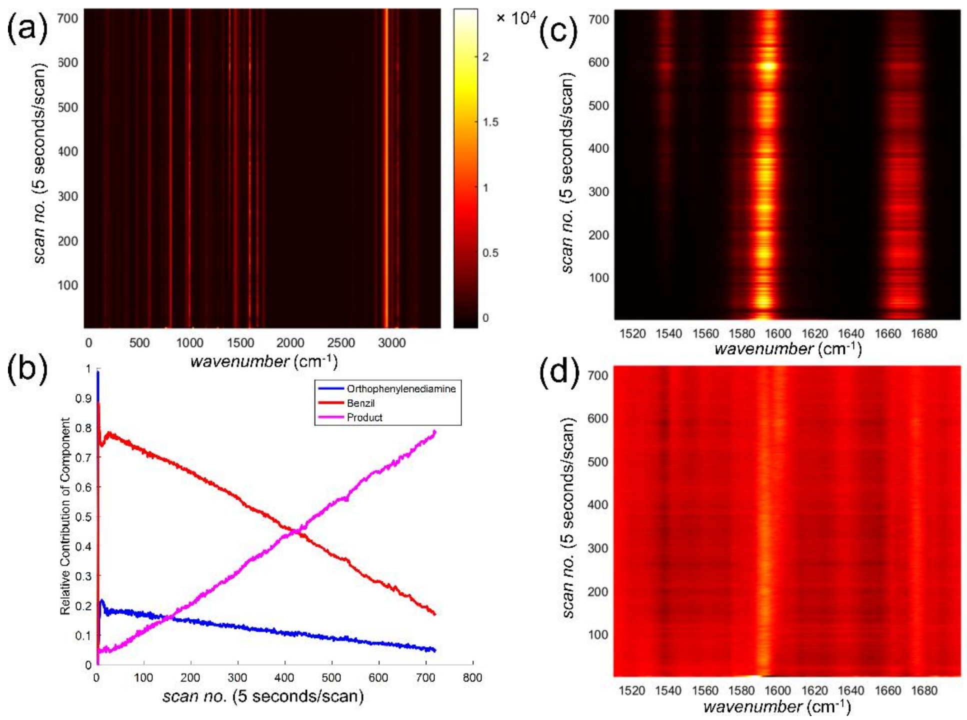

Figure S20: Data for Experiment 2, 20 Hz: (a) the entire time-resolved Raman spectrum for the second experiment conducted by milling at 20 Hz; (b) the associated time-dependent change in spectral contributions of the product (purple) and the two reactants (red and blue); (c) section of the spectrum selected for least-squares fitting and (d) the final residual spectrum.

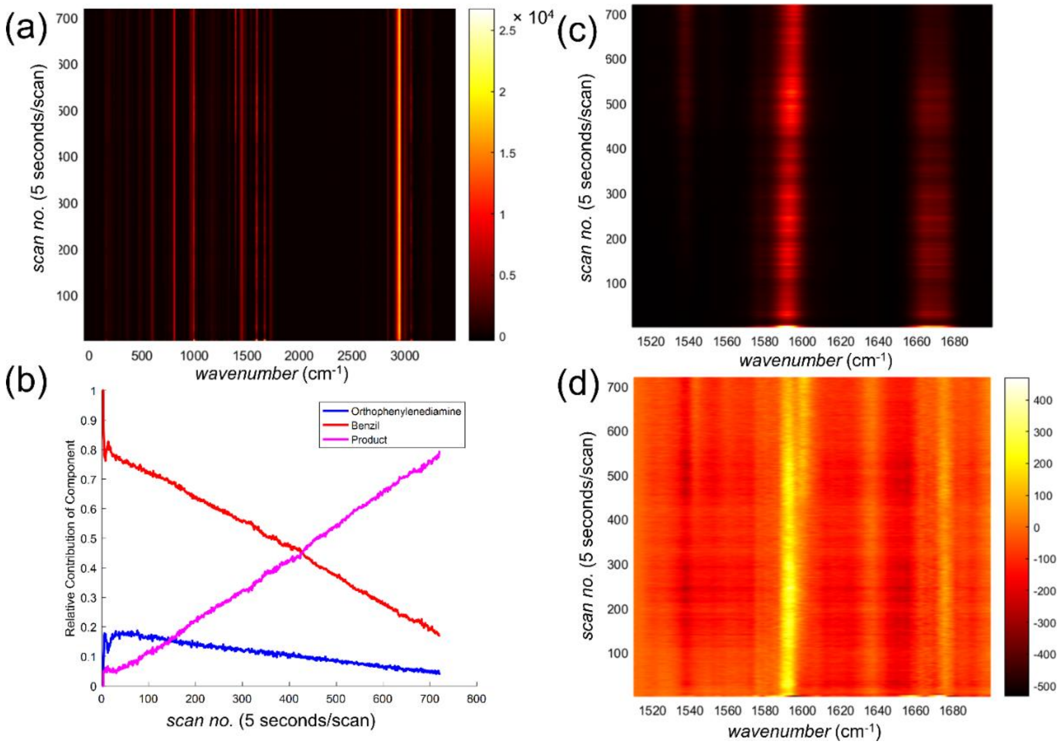

Figure S21: Data for Experiment 3, 20 Hz: (a) the entire time-resolved Raman spectrum for the third experiment conducted by milling at 20 Hz; (b) the associated time-dependent change in spectral contributions of the product (purple) and the two reactants (red and blue); (c) section of the spectrum selected for least-squares fitting and (d) the final residual spectrum.
